# Safety and Performance of INVICTA DF4 Ventricular Defibrillation Lead: Results From APOLLO Prospective Multicenter Study

**DOI:** 10.1111/pace.70286

**Published:** 2026-05-16

**Authors:** Klaus K. Witte, Juan Gabriel Martínez, Mário M. Oliveira, Alberto Barrera, Maite Izquierdo, Cosimo Mandurino, Maria Emilce Trucco, José Juan García Salvador, Antoine Dompnier, Frédéric Jean, Pablo J. Sánchez Millán, Carsten Israel, Maria Martino, Pedro Marques

**Affiliations:** ^1^ Medizinische Klinik I Kardiologie Pneumologie Angiologie Und Internistische Intensivmedizin Universitatsklinikum Aachen Aachen North Rhine‐Westphalia Germany; ^2^ Leeds Institute of Cardiovascular and Metabolic Medicine Faculty of Medicine and Health University of Leeds Leeds West Yorkshire UK; ^3^ Cardiology Department, Arrhythmia Unit Hospital General Universitario Dr Balmis Servicio de Cardiologia Alicante, Valencian Community Spain; ^4^ Cardiology Department, Arrhythmia Unit Unidade Local de Saude Sao Jose Lisbon Portugal; ^5^ Cardiology Department, Arrhythmia Unit Hospital Universitario Virgen De La Victoria Málaga Andalusia Spain; ^6^ Cardiology Department, Arrhythmia Unit Hospital Universitari i Politecnic La Fe Valencia, Valencian Community Spain; ^7^ Cardiology Unit Ospedale SS Annunziata Taranto Taranto Italy; ^8^ Cardiology Department, Arrhythmia Unit Hospital Universitari de Girona Doctor Josep Trueta Girona Catalonia Spain; ^9^ Cardiology Department, Arrhythmia Unit Hospital Universitario de Gran Canaria Dr Negrin Las Palmas de Gran Canaria, Canary islands Spain; ^10^ Service De Cardiologie Centre Hospitalier Annecy Genevois Epagny Metz‐Tessy, Auvergne‐Rhône‐Alpes France; ^11^ Service De Cardiologie médicale et Médecine Vasculaire Centre Hospitalier Universitaire Clermont‐Ferrand Clermont‐Ferrand, Auvergne‐Rhône‐Alpes France; ^12^ Cardiology Department, Arrhythmia Unit Hospital Universitario Virgen De Las Nieves Servicio De Cardiologia Granada Andalusia Spain; ^13^ Klinik Für Innere Medizin – Kardiologie, Diabetologie und Nephrologie Evangelisches Klinikum Bethel GgmbH Bieflefeld North Rhine‐Westphalia Germany; ^14^ MicroPort CRM Saluggia Italy; ^15^ Cardiology Department, Arrhythmia Unit Unidade Local de Saude Santa Maria Lisbon Portugal

**Keywords:** active‐fixation, defibrillation leads, quadripolar DF4 leads, ventricular arrhythmia

## Abstract

**Background:**

Implantable cardioverter‐defibrillator (ICD) therapy relies on reliable right ventricular leads for pacing, sensing, and defibrillation. The INVICTA DF4 lead is an active‐fixation, quadripolar design intended to optimize implant workflow and long‐term safety and performance.

**Methods:**

APOLLO was a prospective, multicenter, single‐arm, observational study enrolling adults across Europe with guideline‐based indications for de novo ICD or Cardiac Resynchronization Therapy Defibrillator (CRT‐D) implantation who received an INVICTA DF4 lead (single‐ or dual‐coil; 58 or 68 cm). The co‐primary endpoints focused on safety and performance at 3 months, while secondary endpoints included long‐term electrical variables, arrhythmia termination, and lead‐related complications through 24 months.

**Results:**

Between June 4, 2021, and October 19, 2022, 446 patients were implanted across 45 sites in 8 European countries. The INVICTA lead was successfully implanted in 100% of the 446 enrolled patients. Leads were positioned either in the apex in 57.2% or right ventricle (RV) septum in 42.8%. Electrical variables stabilized after discharge and remained stable up to 24 months (M24 mean pacing threshold 0.85 ± 0.33 V at 0.5 ms; sensing 13.39 ± 3.17 mV; pacing impedance 525.5 ± 94.2 Ohms; right ventricular (RV) coil continuity 427.8 ± 82.7 Ohms). Of 86 arrhythmic episodes requiring defibrillation, 97.7% were successfully terminated. Lead‐related complication free rate was 97.5% at 30 days, 98.9% at 31–730 days, and 96.4% overall. Mortality was 8.5% (38/446) at two years, of which 63% was due to cardiovascular causes.

**Conclusions:**

In a large, contemporary European cohort, the INVICTA DF4 ventricular lead demonstrated high implant success, durable electrical performance, effective arrhythmia termination, and low complication rates through 24 months. A trend toward less complication in septal positioning was observed compared to apical placement, with comparable electrical performance.

## 1 Introduction

Over the last decades, transvenous Implantable Cardiac Defibrillators (ICD) have become established as an essential part of the optimal management of people at high risk of life‐threatening ventricular arrhythmia. The right ventricular (RV) defibrillation lead plays a critical role in the vast majority of ICD systems. Despite some minor differences in structure, most manufacturers use similar material and equivalent design with respect to pacing, sensing and defibrillation functions.

The DF4 connector, now the industry standard, has largely replaced the trifurcating DF1 design [1], consolidating the connection to the device into a single four‐contact port. This redesign simplifies connector engagement (requiring only one set screw), minimizes early post‐implant connector mismatches and lead abrasion, and reduces both pocket material and procedural time.

The INVICTA defibrillation leads (MicroPort CRM) are active‐fixation, quadripolar DF4 leads intended for use with ICD and Cardiac Resynchronization Therapy Defibrillator (CRT‐D) systems. The safety and performance of INVICTA Ventricular DF4 Leads with Active Fixation (APOLLO) study was a multicenter, pre‐market study conducted to evaluate the safety, electrical performance, and therapy effectiveness of the INVICTA leads. Its primary safety and efficacy results supported the product's CE mark (certificate No. G70 005178 0045 Rev 00, July fifth, 2022).

## 2 Methods

### 2.1 Study Design and Population

APOLLO (S**a**fety and **p**erformance **o**f INVICTA Ventricu**l**ar DF4 **L**eads with Active Fixati**o**n) was a pre‐market, prospective, multicenter, single‐arm European study (CIV‐PT‐20‐07‐033804; NCT04590144), conducted in two sequential phases: Phase I (157 subjects, 3 months follow‐up), and Phase II (446 subjects, 24 months follow‐up). Progression between phases required meeting predefined safety and performance criteria reviewed by an independent Data Safety Monitoring Board (DSMB).

Adults with ESC guidelines [2] indications for de novo ICD or CRT‐D implantation and fulfilling none of the exclusion criteria were eligible to participate. Visits were scheduled at discharge, 1 month, 3 months, and every 6 months through 24 months, in‐clinic or by remote monitoring. The study was approved by national health authorities, local ethics committees, and institutional review boards and all patients provided written informed consent. Safety and performance data were regularly reviewed by the DSMB throughout the study.

### 2.2 Lead Design

INVICTA defibrillation leads are available as single‐coil (1CR) or dual‐coil (2CR) models and in two lengths (58 cm and 68 cm). The lead features an extendable/retractable helix for right ventricle (RV) active fixation and a DF4 quadripolar connector supporting pacing, sensing, and defibrillation and also a silicone soft tip in order to minimize pressure on the cardiac wall. Signal transmission uses microcable technology (stranded MP35N with silver core wires, Ethylene Tetra Fluoro Ethylene (ETFE)‐coated) and MP35N inner‐coil technology with Platinum/Iridium (Pt/Ir) electrodes. Insulation consists of 7.8 F polyurethane multilumen tubing with an outer jacket. All models are steroid‐eluting (330 µg dexamethasone sodium phosphate (DSP)) to reduce inflammation and its associated acute increases in pacing thresholds. The lead design is shown in Figure 1.

**FIGURE 1:**
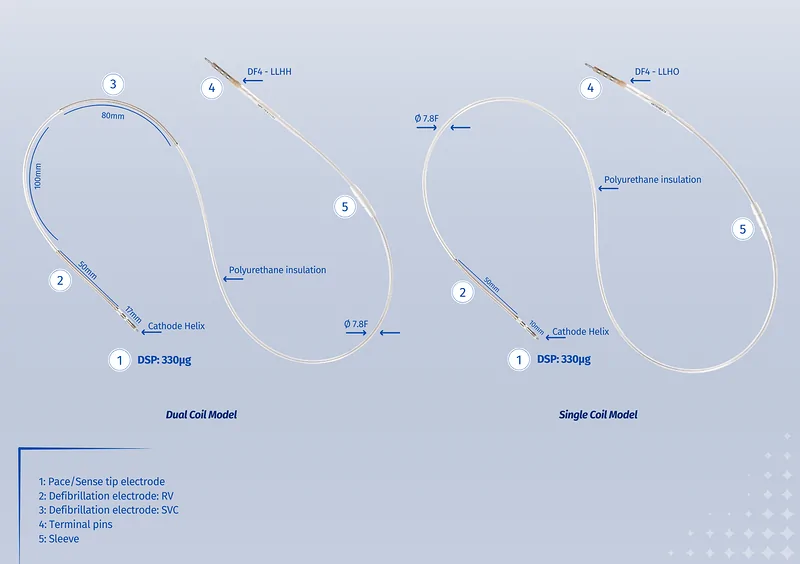
INVICTA lead design—dual coil and single coil models. [Colour figure can be viewed at wileyonlinelibrary.com]

### 2.3 Endpoints and Definitions

The primary objective was safety and performance of the INVICTA lead at 3 months at the end of Phase I including two co‐primary tested endpoints: a co‐primary safety endpoint on the freedom from RV lead‐related complications that occurred up to 91 days from this attempt to implant the INVICTA lead and a co‐primary performance endpoint on the INVICTA manual pacing threshold at 3 month post‐implant. Secondary objectives assessed at 24 months included long‐term lead‐related complications, trends in electrical parameters, arrhythmia termination efficacy, implant handling and success, and adverse events throughout 24 months. The implanters evaluated the lead's handling characteristics and accessories (like helix mechanism, lead advancement in vessels, lead positioning, manoeuvrability, stylets, etc.) using a 4 points scale from Good to Very Poor. Lead‐related complications were defined as serious adverse events at least probably attributable to the lead or its implant procedure that resulted in death or required an additional invasive intervention. Intraoperative RV lead damage or dislodgement and events caused by Twiddler's syndrome were excluded.

Deaths (all‐cause) and lead‐related complications were adjudicated by an independent Clinical Event Committee. Statistical analyses were prespecified in the protocol and Statistical Analysis Plan and performed using SAS 9.4.

## 3 Results

### 3.1 Population and Implant Characteristics

A total of 446 patients were enrolled into the APOLLO study at 45 sites in 8 European countries between June 4, 2021, and October 19, 2022. The inclusion criteria for patients were: (1) any patient presenting an ICD or CRT‐D indication as detailed in the latest ESC guidelines, (2) scheduled for a de‐novo implant of an ICD (VR, DR) or CRT‐D, manufactured by MicroPort CRM and equipped with a DF4 connector and (3) signed and dated informed consent. The exclusion criteria were the following: tricuspid valvular disease or any type of tricuspid replacement heart valve (mechanical or tissue), transient tachyarrhythmias due to reversible causes (such as drug intoxication, electrolyte imbalance, sepsis, hypoxia or other factors as myocardial infarction or electric shock), contraindication to a maximum single dose of 330 µg DSP, active myocarditis, previous implant of pacemaker, ICD or CRT‐D device and leads, currently enrolled or planning to participate in a potentially confounding drug or device trial during the course of this study, incapacitated subject or under guardianship, inability to understand the purpose of the study or to meet follow‐up visits at the implanting centre as defined in the investigational plan, minor subjects, pre‐menopausal women, drug addiction or abuse, life expectancy less than 1 year. The cohort comprised 85% males with a mean age of 66.4 years. Ischemic cardiomyopathy was present in 55.8%, hypertension in 66.8%, diabetes in 36.5%, and renal insufficiency in 14.1%. Atrial fibrillation was present in 14.0% of subjects. Three‐hundred and seventy‐three (373) patients received an ICD (single‐chamber 51.6%, dual‐chamber 32.0%), and 73 (16.4%) received a CRT‐D device (all devices manufactured by MicroPort CRM). Primary prevention accounted for 75.6% of implants. Venous access was subclavian in 43.3%, cephalic in 38.8%, and axillary in 17.9% of the patients. Mean left ventricular ejection fraction (LVEF) was 35.2  ±  13.1% and mean QRS duration was 108.0  ±  24.3 ms in the VR/DR population, compared with 29.1  ±  8.5% and 162.3  ±  29.2 ms, respectively, in the CRT‐D population (Table 1).

**TABLE 1:** Baseline characteristics of the enrolled population.

	Device type		INVICTA lead position		ALL (*N* = 446)
	ICD (*N* = 373)	CRT‐D (*N* = 73)	Apex (*N* = 255)	Septum (*N* = 191)	
**Male** *N* (%)	315 (84.5)	64 (87.7)	213 (83.5)	166 (86.9)	379 (85.0)
**Age** mean (SD)	65.8 (10.4)	69.8 (9.8)	66.2 (10.5)	66.6 (10.3)	66.4 (10.4)
**BMI** mean (SD)	27.3 (4.9)	27.3 (5.2)	27.6 (5.2)	26.8 (4.5)	27.8 (4.9)
**Cardiomyopathy** *N*(%)	315 (84.5)	69 (94.5)	228 (89.4)	156 (81.7)	384 (86.1)
Ischemic	190 (50.9)	26 (35.6)	136 (53.3)	80 (41.9)	216 (48.4)
Dilated	54 (14.5)	29 (39.7)	43 (16.9)	40 (20.9)	83 (18.6)
Hypertrophic	27 (7.2)	1 (1.4)	19 (7.5)	9 (4.7)	28 (6.3)
Ischemic & dilated	25 (6.7)	8 (11.0)	20 (7.8)	13 (6.8)	33 (7.4)
Valvular	2 (0.5)	3 (4.1)	2 (0.8)	3 (1.6)	5 (1.1)
Hypertensive	1 (0.3)	0 (0.0)	0 (0.0)	1 (0.5)	1 (0.2)
Hypertrophic & Dilated	0 (0.0)	1 (1.4)	0 (0.0)	1 (0.5	1 (0.2)
Other	15 (4.0)	1 (1.4)	8 (3.1)	8 (4.2)	16 (3.6)
**LBBB** *N* (%)	24 (6.4)	58 (79.5)	43 (16.9)	39 (20.4)	82 (18.4)
**Renal insufficiency** *N* (%)	45 (12.1)	18 (24.7)	34 (13.3)	29 (15.2)	63 (14.1)
**Myocardial infarction** *N* (%)	207 (55.5)	22 (30.1)	136 (53.3)	93 (48.7)	229 (51.3)
**Diabetes mellitus** *N* (%)	128 (34.3)	35 (47.9)	103 (40.4)	60 (31.4)	163 (36.5)
**Sinus rhythm** *N* (%)	322 (87.0)	59 (80.8)	—	—	381 (86.0)
**Atrial Fibrillation** *N* (%)	48 (13.0)	14 (19.2)	—	—	62 (14.0)
**Average HR** mean (SD)	69.33 (12.93)	71.03 (16.07)	—	—	69.60 (13.49)
**QRS** mean (SD)	108.02 (24.30)	162.30 (29.20)	—	—	117.17 (32.36)
**LVEF** *N* (%)	35.27 (13.04)	29.11 (8.54)	—	—	34.24 (12.61)
**NYHA class** I *N* (%)	87 (23.6)	3 (4.2)	—	—	90 (20.4)
II	227 (61.5)	48 (66.7)	—	—	275 (62.4)
III	51 (13.8)	21 (29.2)	—	—	72 (16.3)
IV	4 (1.1)	0 (0.0)	—	—	4 (0.9)
**CRT‐D Device** *N* (%)	—	—	36 (14.1)	37 (19.4)	73 (16.4)

Implant success was 100%, with first‐attempt success in 98.4% (339/446 patients). In 7 patients, a second INVICTA lead was used during the same implantation procedure due to misuse in 6 patients and sensing issue in 1 patient. Lead type (97.3% single vs. 2.7% dual coil) and length (57.4 % with 58 cm or 42.6% with 68 cm) were chosen according to the implanters’ preference with a view to patient variables. The lead was predominantly positioned in the apex (57.2% vs. 42.8% in the RV septum). The implanters' assessment of lead handling and accessories was rated Good in 95.5% and 90.5% of the cases, respectively.

The mean average follow‐up duration was 696.5 ± 176.9 days (median: 746.5 days).

### 3.2 Primary Endpoints

The co‐primary endpoints of safety and performance were evaluated in the first 157 enrolled patients at the 3‐month follow‐up. The proportion of subjects free from lead–related complications up to 91 days post‐implant was 97.40% (95% CI: 93.22%–99.02%). The manual pacing threshold at 0.50 ms was 0.86 ± 0.26 V (95% CI: 0.82–0.91 V; *p* < 0.0001). Given the prespecified performance goals of ≥ 90% complication‐free rate and a pacing threshold < 1.25 V at 0.50 ms, both co‐primary endpoints were successfully met.

### 3.3 Secondary Endpoints

#### 3.3.1 Electrical Performance Through 24 Months

The electrical parameter trends from implant to M24 visits are reported in Figure 2 for manual threshold analysis measured at 0.5 ms, sensing amplitude, pacing impedance and RV coil continuity impedance.

**FIGURE 2:**
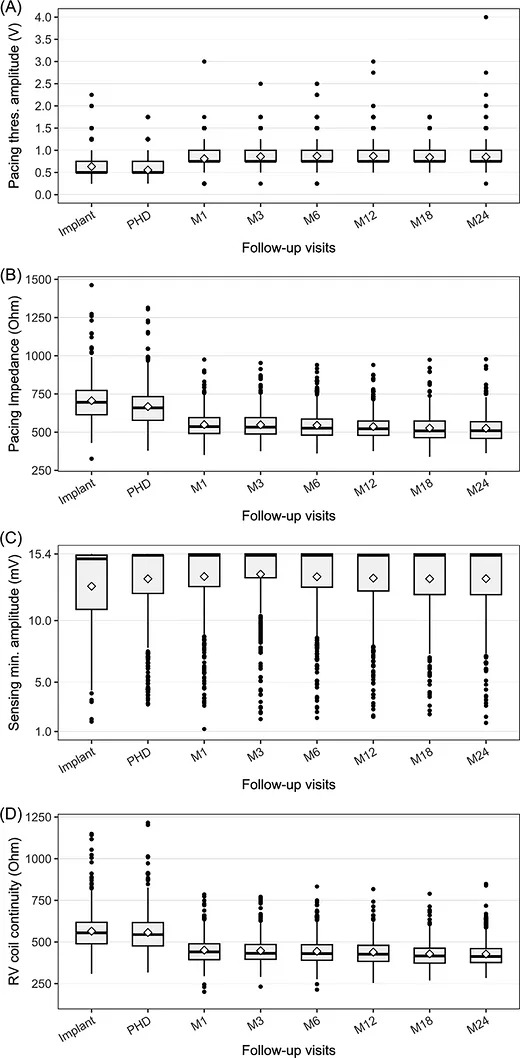
Box plots for INVICTA lead (A) Manual pacing threshold amplitude at 0.5 ms (V) (B) Minimum pacing impedance (Ohms) (C) Sensing minimum amplitude (mV) (D) RV coil continuity (impedance) (Ohms) by visit.

Overall, the electrical parameters stabilized post‐discharge and remained unchanged throughout the two‐year follow‐up period. At M24, the mean pacing threshold was 0.85 ± 0.33 V, pacing impedance 525.5 ± 94.2 Ohms, sensing threshold 13.39 ± 3.17 mV and RV coil continuity 427.8 ± 82.7 Ohms.

#### 3.3.2 Efficacy of Ventricular Arrhythmia Termination

From implant to study end, 86 ventricular arrhythmia episodes requiring shocks occurred in 39 patients (8.7%). Six episodes of induced ventricular fibrillation at implant were all successfully terminated with a mean delivered energy of 24.6 J. The remaining 80 spontaneous episodes occurred in 33 patients of which 78 (97.5%) were successfully treated (mean delivered energy 37 J). Two shocks were ineffective in one patient with Brugada syndrome, with spontaneous recovery after two episodes and successful termination of a third. Overall, effective shock therapy was delivered in 97.7% of episodes.

#### 3.3.3 Lead‐Related Complications

Kaplan–Meier‐estimated freedom from INVICTA lead‐related complications was 97.53% acute (≤ 30 days), 98.85% chronic (31–730 days), and 96.39% overall from implant to study end (Figure 3). Sixteen patients (3.6%) experienced 17 complications: 10 lead dislodgements, 5 losses of capture, and 2 cardiac perforations. The majority of complications occurred within the first month post‐implantation (*n* = 11), with an additional five events reported between days 31 and 66. No further complications were observed from day 67 to day 730. (Figure 3).

**FIGURE 3:**
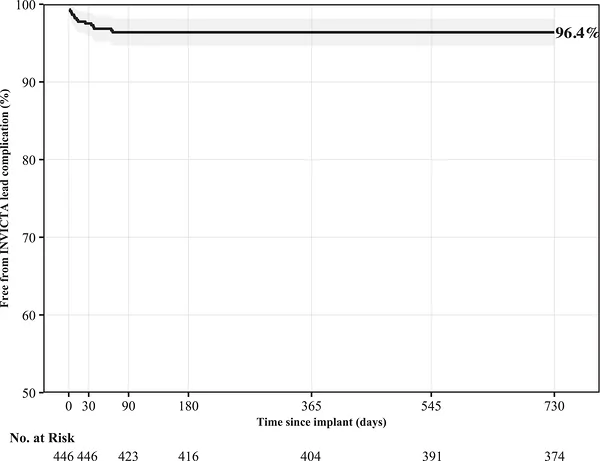
INVICTA lead‐related complication‐free survival—confidence interval in grey zone.

There were another 14 events conservatively assessed as possibly related to the INVICTA lead or its implantation, but not adjudicated as complications, included Twiddler's syndrome, procedure‐related dislodgement, oversensing, thrombosis, pocket hematoma/infection, cardiogenic and septic shock, ventricular arrhythmia at implant, ventricular fibrillation, and unknown cause of death. There were no unexpected serious adverse effects related to the device under investigation.

#### 3.3.4 All‐Cause Mortality Rates

Thirty‐eight subjects (8.5%) died from any cause during the study, 4 deaths occurred during the first month after implantation, 15 within the first year, 15 between months 12 and 18, and 4 thereafter. Of these, 24 deaths (63%) were attributed to cardiovascular causes, 10 to non‐cardiovascular causes, and in 4 cases the cause remained undetermined. There was no statistically significant difference in mortality between apical and septal lead positions (9.4% vs. 7.3%, *p* = 0.54).

#### 3.3.5 Apical Versus RV Septal Positioning

Baseline characteristics and device type distribution (ICD vs. CRT‐D) were similar between patients implanted with the INVICTA lead in apical (*N* = 255) versus RV septal (*N* = 191) positions (Table 1). No significant differences were also observed between apical and septal groups in pacing thresholds (0.84 ± 0.33 V vs. 0.88 ± 0.39 V at M24, respectively), pacing impedances (524.7 ± 90.9 Ohms vs. 526.5 ± 98.6 Ohms), sensing amplitudes (13.84 ± 2.73 mV vs. 12.83 ± 3.58 mV), and coil impedances (432.5 ± 85.4 Ohms vs. 421.9 ± 78.9 Ohms) over time. Nevertheless, the complication‐free rate was higher for septal implants (98.4%) than apical implants (94.9%) (Figure 4). Septal complications consisted of lead dislodgement (*n* = 2) and loss of capture (*n* = 1), whereas apical complications included dislodgement (*n* = 8), loss of capture (*n* = 4), and cardiac perforation (*n* = 2).

**FIGURE 4:**
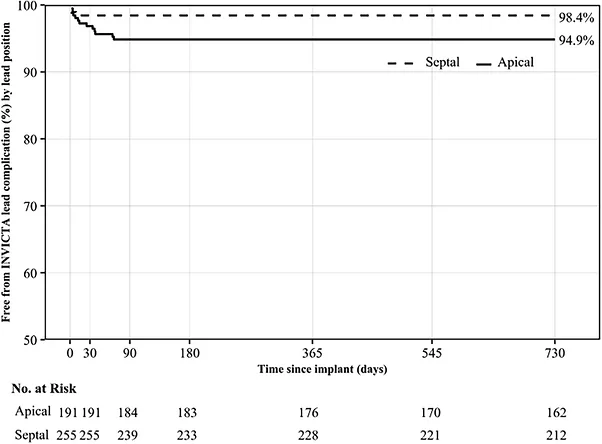
INVICTA lead‐related complication‐free survival by RV lead position.

## 4 Discussion

The APOLLO prospective investigation demonstrated the safety and performance of the INVICTA DF‐4 quadripolar active fixation defibrillation lead: out of the 446 patients implanted with the INVICTA lead and followed up to 24 months, 2.5% of patients presented lead complications in acute phase (≤ 30 days) and 1.1% of patients in chronic phase (from 31 days up to 730 days). Electrical variables were as expected and remained stable throughout the two‐year follow‐up period, with no significant difference between apical and septal placement. Septal placement was associated with fewer complications than apical placement.

ICD leads, when connected to an Implantable Cardiac Defibrillator, are intended for automated treatment of life‐threatening ventricular arrhythmias via ventricular anti‐tachycardia pacing (ATP) and defibrillation, thus preventing Sudden Cardiac Arrest [3], and for the treatment of bradycardia via ventricular pacing.

The DF4 connector is the industry standard for the connection of a defibrillator lead to the pulse generator. With the DF4 connector, instead of three separate ports to connect to the RV‐ lead, there is a single port with four contacts, including high voltage for shock delivery. In previous studies, leads equipped with DF4 connector demonstrated the high safety profile of such lead design in both acute (until 3 months) and chronic phase (after 3 months).

The APOLLO study is a multicenter clinical trial, which aimed to demonstrate the safety and performance of the INVICTA quadripolar DF4 active fixation defibrillation lead. APOLLO confirmed a high rate of freedom from lead‐related complications, with 97.5% at 30 days and 98.9% from 31 days up to two years, resulting in an overall 96.4% complication‐free survival. Interestingly, the complications appeared early, and no new complications occurred beyond 66 days post‐implant. There were no unexpected serious adverse effects related to the INVICTA lead throughout the study.

Available data from the scientific literature on contemporary ICD leads equipped with a DF‐4 connector indicate that lead‐related complications remain an important safety consideration in clinical practice. These complications may involve electrical abnormalities or mechanical issues affecting the integrity and performance of the lead. Reported adverse events include abnormal defibrillation impedance, elevated pacing thresholds, failure to appropriately detect ventricular tachycardia (VT) or ventricular fibrillation (VF), lead dislodgement, lead fracture, loss of capture, and cardiac perforation. Several clinical studies evaluating contemporary ICD leads (including the St. Jude Medical (Abbott) Durata 7122Q, the Medtronic Sprint Quattro Secure 6935 M, and the Boston Scientific Endotak Reliance 4‐Site (0293) and 4‐Front (0693) leads) have documented the incidence and timing of lead‐related complications in real‐world populations. In a multicenter cohort among 812 implanted ICD leads including these models, Sarrazin et al. reported lead‐related complications in 4.4 % of patients within 3 years [4]. Similarly, other observational studies evaluating modern transvenous ICD leads have described cumulative lead failure or complication rates ranging from 2.9% to 4.5% during long‐term follow‐up depending on population characteristics and duration of observation [5]. Therefore, the results of APOLLO, with 3.6% of complications over long‐term follow‐up period up to 24 months are comparable with the published data with safety and reliability comparable to similar devices on the market. The mortality rate of 8.5% at 2 years was also within the expected range for populations implanted with an ICD [6, 7, 8].

Evidence from clinical registries and observational studies also indicates that the majority of such complications occur during the early post‐implantation period, typically within the first weeks to months after the procedure [4, 6]. The present results are in line with this, showing that 11 patients had lead complications that occurred within 30 days from implant and that an additional 5 patients had complications between 31 days and 66 days from implant. No additional complications occurred until the end of follow‐up (730 days from implant).

In addition, no difference in electrical performance was observed in APOLLO between septal and apical placement, although the complication‐free rate was higher for septal lead placement (98.4%) compared to apical placement (94.9%). These results also align with growing evidence showing that septal lead placement is considered an acceptable and possibly preferred alternative to apical pacing in terms of safety depending on the patient's profile (mechanics, perforation risk, dyssynchrony) [9, 10, 11]. However, it should be noted that current recommendations still acknowledge that septal pacing is not mandated over apical pacing due to the lack of overwhelming evidence of functional superiority and the technical challenge that this requires [12, 13].

Finally, APOLLO demonstrated the efficacy of INVICTA leads in terminating VT and/or VF with high success rate of shock therapy for ventricular arrhythmias (97.5% for spontaneous episodes) indicating reliable energy delivery and supporting the lead's capacity to effectively terminate life‐threatening arrhythmias, thereby contributing to patient safety. The few ineffective shocks, observed in a single patient with Brugada syndrome, highlight the importance of individualized management in patients with complex arrhythmic substrates.

## 5 Limitations

Event adjudication focused on lead‐related complications and all‐cause mortality; device/system factors or clinical confounders may not have been fully considered. Despite multicenter enrollment, site‐specific implant practices and experience could have influenced some outcomes, for example the rate of acute complications post‐implantation. However, the aim was to explore the safety and utility of the lead in usual care. Also, although follow‐up extended to 24 months, very long‐term performance beyond two years was not assessed.

## 6 Conclusions

The INVICTA DF4 RV lead exhibited high implant success, stable electrical variables, effective arrhythmia termination, and low complication rates through two years in a large, multicenter European cohort. There is a trend toward less complication in septal positioning compared to apical placement while maintaining comparable electrical performance. These results support the INVICTA DF4 lead as a reliable option for ICD and CRT‐D therapy in routine clinical practice.

## Author Contributions

Dr K.K Witte reviewed and revised the manuscript draft. All authors reviewed and approved the final manuscript.

## Funding

This study was funded by MicroPort CRM, France.

## Ethics Statement

The study has been conducted in accordance with the ethical principles that have their origin in the Declaration of Helsinki, and that are consistent with Good Clinical Practice described in ISO 14155 and the applicable regulatory requirement(s) of the countries where the study is conducted. The study has been registered on the ClinicalTrials.gov website (NCT04590144) and updated on a regular basis. Signed patient informed consent was obtained by the investigator or delegated person, prior to enrolling the patient in the study.

## Conflicts of Interest

Maria Martino is employed by MicroPort CRM, Saluggia, Italy. Dr. K.K. Witte received compensation for his assistance with the manuscript preparation. The authors received a fee from MicroPort CRM for participation in this study. The authors declare no other conflicts of interest.

## Data Availability

The data supporting the findings of this study are available from the corresponding author upon reasonable request. The data are not publicly available due to privacy or ethical restrictions.

## APPENDIX 1 APOLLO—Study Committees, Boards, and Investigators

### 1.1

**Independent Data Safety Monitoring Board**: Pr Jean‐Claude Deharo—CHU de Marseille—Hôpital de la Timone—Marseille‐ France; Dr David Sharman—Northampton General Hospital‐ Northampton United Kingdom; Dr Elvis Teijeira Fernandez—Complejo Hospitalario Universitario de Vigo, Spain. **Clinical Event Committee**: Dr Marcello Piacenti—Fondazione Toscana G. Monasterio, C.N.R. Pisa, Italy. **APOLLO Trial Investigators: *Austria***: Dr. Clemens Steinwender‐ Kepler Universitätsklinikum GmbH. Linz; ***Germany***: Dr. Klaus Witte—RWTH Aachen. Aachen; Dr. Peter Falk—Herz‐ und Gefäßzentrum Bad Bevensen. Bad Bevensen; Dr. Carsten Israel—Evangelisches Klinikum Bethel. Bielefeld; Dr. Thomas Faber—Universitäts‐Herzzentrum Freiburg ‐Bad Krozingen. Freiburg im Breisgau; Dr. Christoph Axthelm—Hausärztlich‐Kardiologisches MVZ “Am Felsenkeller” GmbH. Pirna; ***France***: Prof. Jacques Mansourati—CHU Brest. Brest; Dr. Antoine Dompnier—CH Annecy. Metz‐Tessy; Dr. Frédéric Jean—CHU Clermont‐Ferrand. Clermont‐Ferrand; Prof. Pascal Defaye—CHU Grenoble. La Tronche; Dr. Laurence Guedon‐Moreau—CHU Lille. Lille; Prof. Laurence Jesel—CHU Strasbourg. Strasbourg; Dr. Bertrand Pierre—CHU Tours. Chambray Les Tours; Dr. Edouard Gitenay—Hôpital St Joseph. Marseille; ***Great Britain***: Dr. Joseph Tomson—Barnet Hospital, Royal Free Trust. Barnet; Prof. Francisco Leon Leyva—Queen Elizabeth. Birmingham; Dr. Badrinathan Chandrasekaran—Great Western Hospital. Swindon; ***Italy***: Dr. Eduardo Celentano—Cliniche Humanitas Gavazzeni. Bergamo; Dr. Giosuè Mascioli—Ospedale di Desenzano del Garda. Montecroce BS; Dr. Domenico Facchin—AO Univ. S. Maria della Misericordia. Udine; Dr. Giuseppe Picciolo—Ospedale Piemonte (IRCCS Bonino Pulejo). Messina; Dr. Antonio Ruocco—AO di Rilievo Nazionale A. Cardarelli. Napoli; Dra. Anna Rago—Univ. degli Studi della Campania L. Vanvitelli. Napoli; Dr. Giovanni Luzzi—Osp SS Annunziata. Taranto; Dr. Antonio Rossillo—AULSS 8 Berica—Vicenza. Vicenza; ***Netherlands***: Dr. Abdul Ghani—Isala Klinieken. Zwolle; Dr. Arjen Pronk—Elisabeth Tweesteden Ziekenhuis. Tilburg; ***Portugal***: Dr. Francisco Madeira—ULS Amadora/Sintra. Amadora; Dr. Luis Elvas—ULS de Coimbra. Coimbra; Dr. Pedro Dionisio—ULS Alentejo Central. Evora; Dr. Pedro Marques—ULS Santa Maria. Lisboa; Dr. Victor Sanfins—ULS do Alto Ave. Creixomil; Prof. Mário Oliveira—ULS de Sao Jose—Hospital Santa Marta. Lisboa; Dra. Helena Gonçalves—ULS Gaia/Espinho. Vila Nova de Gaia; Dr. Hipólito Reis ***—*** ULS Santo Antonio. Porto; ***Spain***: Dr. Juan Gabriel Martínez—Hospital General Universitario Dr. Balimis de Alicante. Alicante; Dr. Pablo Sanchez Millan—Hospital Virgen de las Nieves. Granada; Dr. Angel Ferrero—Hospital Clínico Universitario de Valencia. Valencia; Dra. Maite Izquierdo—Hospital Universitario y Politécnico La Fe. Valencia; Dr. Victor Palanca—Hospital General Universitario de Valencia. Valencia; Dr. Alberto Barrera—Hospital Universitario Virgen de la Victoria. Málaga; Dra. María Emilce Trucco—Hospital Universitario de Girona Dr. Josep Trueta. Girona; Dr. Jose Juan Garcia Salvador—Hospital Universitario de Gran Canaria. Las Palmas de Gran Canaria; Dr. Nicasio Pérez‐Castellano—Hospital Clinico de Madrid. Madrid; Dr. Rafael Salguero—Hospital Universitario 12 de Octubre. Madrid.
